# Targeting formyl peptide receptor 2 to suppress neuroinflammation in neuromyelitis optica spectrum disorder

**DOI:** 10.7150/thno.107303

**Published:** 2025-03-19

**Authors:** Caiyun Qi, Hongying Hao, Wei Zhang, Yiwei Fu, Yali Han, Jinyi Li, Lixiang Chen, Guiyun Cui, Qing Liu, Yuan Li, Xiaozhen Wang, Ming-Wei Wang, Qiang Liu

**Affiliations:** 1 Department of Neurology, Parkinson's Disease Center, The Affiliated Hospital of Xuzhou Medical University, Xuzhou, China.; 2 Department of Neurology, Tianjin Neurological Institute, Tianjin Institute of Immunology, State Key Laboratory of Experimental Hematology, International Joint Laboratory of Ocular Diseases, Ministry of Education, Haihe Laboratory of Cell Ecosystem, Laboratory of Post-Neuroinjury Neurorepair and Regeneration in Central Nervous System Tianjin & Ministry of Education, Tianjin Medical University General Hospital, Tianjin 300052, China.; 3 The National Center for Drug Screening, Shanghai Institute of Materia Medica, Chinese Academy of Sciences, Shanghai 201203, China.; 4 Research Center for Deepsea Bioresources, Sanya 572025, China.; 5 Department of Pharmacology, School of Basic Medical Sciences, Fudan University, Shanghai 200032, China.; 6 Research Center for Medicinal Structural Biology, National Research Center for Translational Medicine at Shanghai, State Key Laboratory of Medical Genomics, Ruijin Hospital, Shanghai Jiao Tong University School of Medicine, Shanghai 200025, China.; 7 Engineering Research Center of Tropical Medicine Innovation and Transformation of Ministry of Education, School of Pharmacy, Hainan Medical University, Haikou 570228, China.

**Keywords:** FPR2/mFpr2, demyelination, neuroinflammation, neuromyelitis optica spectrum disorder

## Abstract

**Background:** Neuromyelitis optica spectrum disorder (NMOSD) is an antibody-mediated neurological inflammatory disease. As a G protein-coupled receptor, formyl peptide receptor 2 (FPR2) orchestrates innate and adaptive immunity. Yet the precise role of FPR2 in neuroinflammation is poorly understood.

**Methods:** Peripheral blood samples were collected from patients with NMOSD and healthy controls. Single-cell RNA sequencing (scRNA-seq) and flow cytometry were employed to assess the expression of FPR2 in immune cell subsets. We used a mouse model of NMOSD to examine the therapeutic potential and underlying immune mechanisms of an FPR2 antagonist Quin-C7. MRI and immunostaining were performed to quantify central nervous system injury.

**Results:** ScRNA-seq and flow cytometry analyses revealed that FPR2 was expressed in various myeloid and lymphoid cell types in patients with NMOSD and a mouse model of NMOSD. In NMOSD mice, mouse formyl peptide receptor 2 (mFpr2) was mainly upregulated in microglia. Administration of Quin-C7 led to reduced brain lesion volume, astrocyte loss and demyelination in NMOSD mice. Further, FPR2 antagonism reduced the inflammatory activity of microglia and lymphocyte infiltration into the brain. Notably, depletion of microglia using a CSF1R inhibitor diminished the protective effects of FPR2 antagonism, suggesting that microglia contribute to the benefit of FPR2 antagonism in NMOSD. In contrast, genetic deficiency of T and B cells or antibody depletion of NK cells did not affect the benefit of FPR2 antagonism.

**Conclusion:** Collectively, our findings revealed a previously unrecognized role of FPR2/mFpr2 in control of microglia activity during neuroinflammation, implying that FPR2 antagonism may serve as a viable therapeutic approach to restrict detrimental neuroinflammation and warrant further investigation.

## Introduction

Neuromyelitis optica spectrum disorder (NMOSD) is a debilitating autoimmune demyelinating disorder of the central nervous system (CNS), primarily characterized by recurrent episodes of optic neuritis and longitudinally extensive transverse myelitis. In ~75% of patients with NMOSD, disease progression is driven by the autoantibodies targeting aquaporin-4 (AQP4), a water channel protein predominantly expressed on astrocytes. Binding of these autoantibodies to AQP4 causes antibody-dependent cell-mediated cytotoxicity (ADCC) and complement-dependent cytotoxicity (CDC), leading to astrocyte loss, demyelination and axonal damage^1^. The initiation of this cascade activates microglia^2^, ^3^. As the first responder to signals derived from astrocyte injury, microglia drive subsequent inflammatory responses that expedite damage to neural structures^2^, ^4^. Despite recent advances in NMOSD pathology and the use of immunosuppressants, many NMOSD patients are afflicted by recurrent relapses and neurological impairment, underscoring the urgent need for novel therapeutic approaches for NMOSD^5^, ^6^.

Formyl peptide receptor 2 (FPR2) is a member of the G protein-coupled receptor superfamily that orchestrates innate and adaptive immune responses^7^. As a chemotactic receptor and pattern recognition receptor to recognize bacterial and mitochondria-derived formylated peptides, FPR2 is mainly expressed in myeloid and lymphoid cell subsets. Although the distribution and function indicate FPR2 as a master switch that governs the initiation and progression of inflammatory responses^8^, ^9^, the precise role of FPR2 in neuroinflammation and NMOSD pathology remains poorly understood.

In this study, we examined the expression profile of FPR2 and its mouse counterpart (mFpr2) in various immune cells subsets from human peripheral blood samples collected from patients with NMOSD and its mouse model using single-cell RNA sequencing (scRNA-seq) and flow cytometry. In NMOSD mice, we assessed the expression of mFpr2 in immune cell subsets and determined the role of mFpr2 in CNS demyelination, astrocyte loss and underlying mechanisms using a FPR2 antagonist Quin-C7.

## Results

### Expression profile of FPR2 in immune cell subsets from NMOSD patients

**Figure 1:**
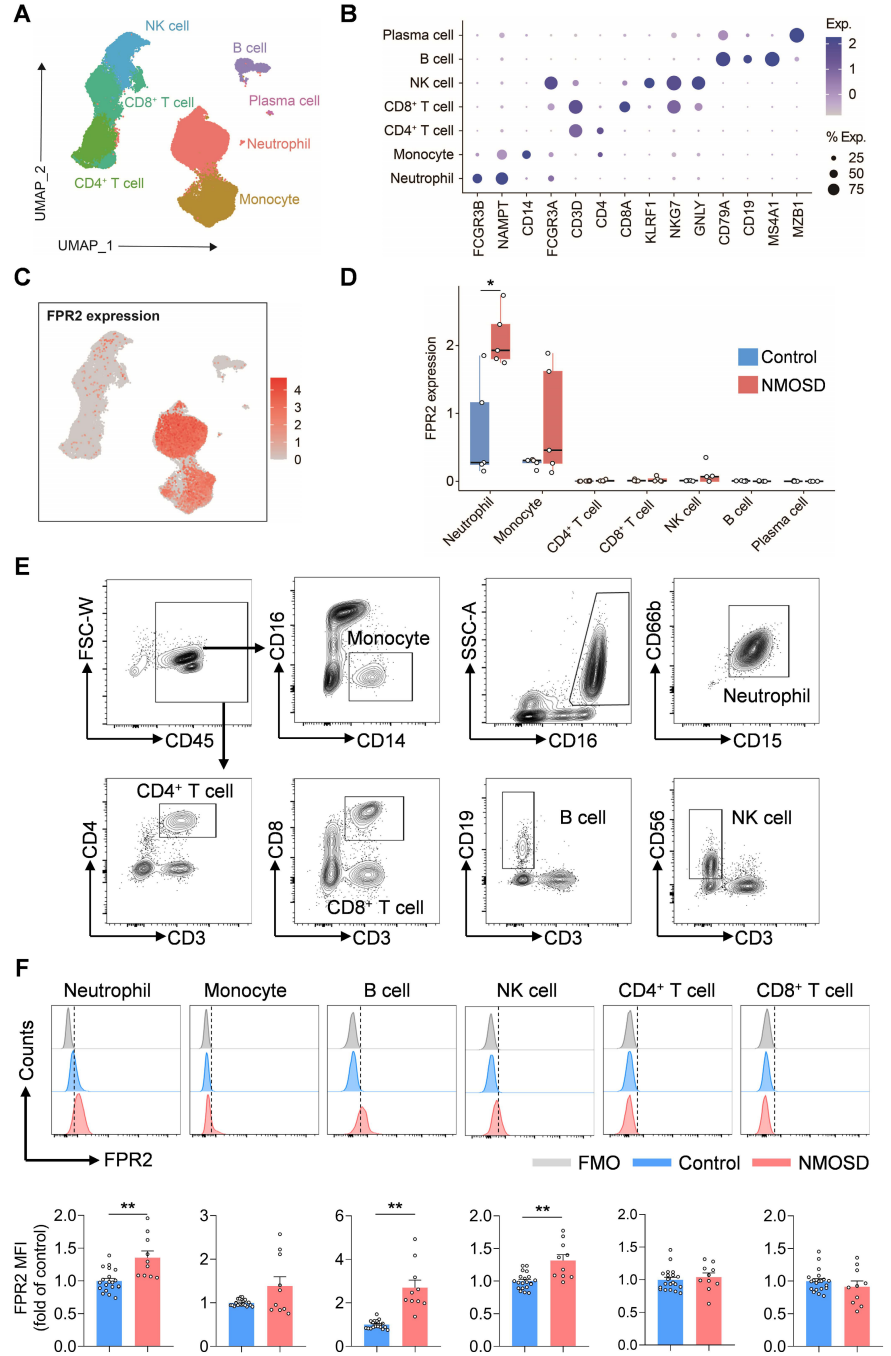
** FPR2 expression profile in immune cell subsets from NMOSD patients.** (**A**) UMAP plot showing the distribution of 90,381 peripheral blood immune cells from 5 patients with NMOSD and 5 control subjects, colors represent distinct cell types. (**B**) Dot plot showing the expression of cell type-specific markers. The dot size and color represent the expression percentage and expression level, respectively. (**C**) The feature plot illustrates the expression of FPR2, with color indicating the expression level. (**D**) Boxplot shows the expression level of FPR2 at individual level. (**E**) Gating strategy of peripheral blood monocytes (CD45^+^CD16^-^CD14^+^), neutrophils (CD45^+^CD16^+^CD15^+^CD66b^+^), CD4^+^ T cells (CD45^+^CD3^+^CD4^+^), CD8^+^ T cells (CD45^+^CD3^+^CD8^+^), B cells (CD45^+^CD3^-^CD19^+^) and NK cells (CD45^+^CD3^-^CD56^+^). (**F**) Histograms showing FPR2-expressing cell subsets in patients with NMOSD and control subjects. Bar graphs show the mean fluorescence intensity (MFI) of FPR2 in neutrophils, monocytes, B cells, NK cells, CD4^+^ T cells and CD8^+^ T cells in NMOSD patients and control subjects. Control: n = 20, NMOSD patients: n = 10. Data are presented as mean ± SEM. *P < 0.05, **P < 0.01. FMO: fluorescence minus one.

To determine the expression profile of FPR2 in various immune cell populations, single-cell suspensions were prepared from peripheral blood samples of five patients with NMOSD and five control subjects for scRNA-seq. Unbiased clustering of 90,381 cells identified seven major cell subsets based on previously reported cell type-specific markers^10^: neutrophils, monocytes, CD4^+^ T cells, CD8^+^ T cells, NK cells, B cells, and plasma cells (**Figure 1A-B**). We found that FPR2 was mainly expressed in neutrophils and monocytes and upregulated in blood neutrophils following NMOSD (**Figure 1C-D**). Similarly, flow cytometry analysis revealed the expression of FPR2 in various immune cell subsets, along with an increase in neutrophils, B cells, and NK cells from patients with NMOSD (**Figure 1E-F**).

### Expression profile of mFpr2 in immune cell subsets from a NMOSD mouse model

**Figure 2:**
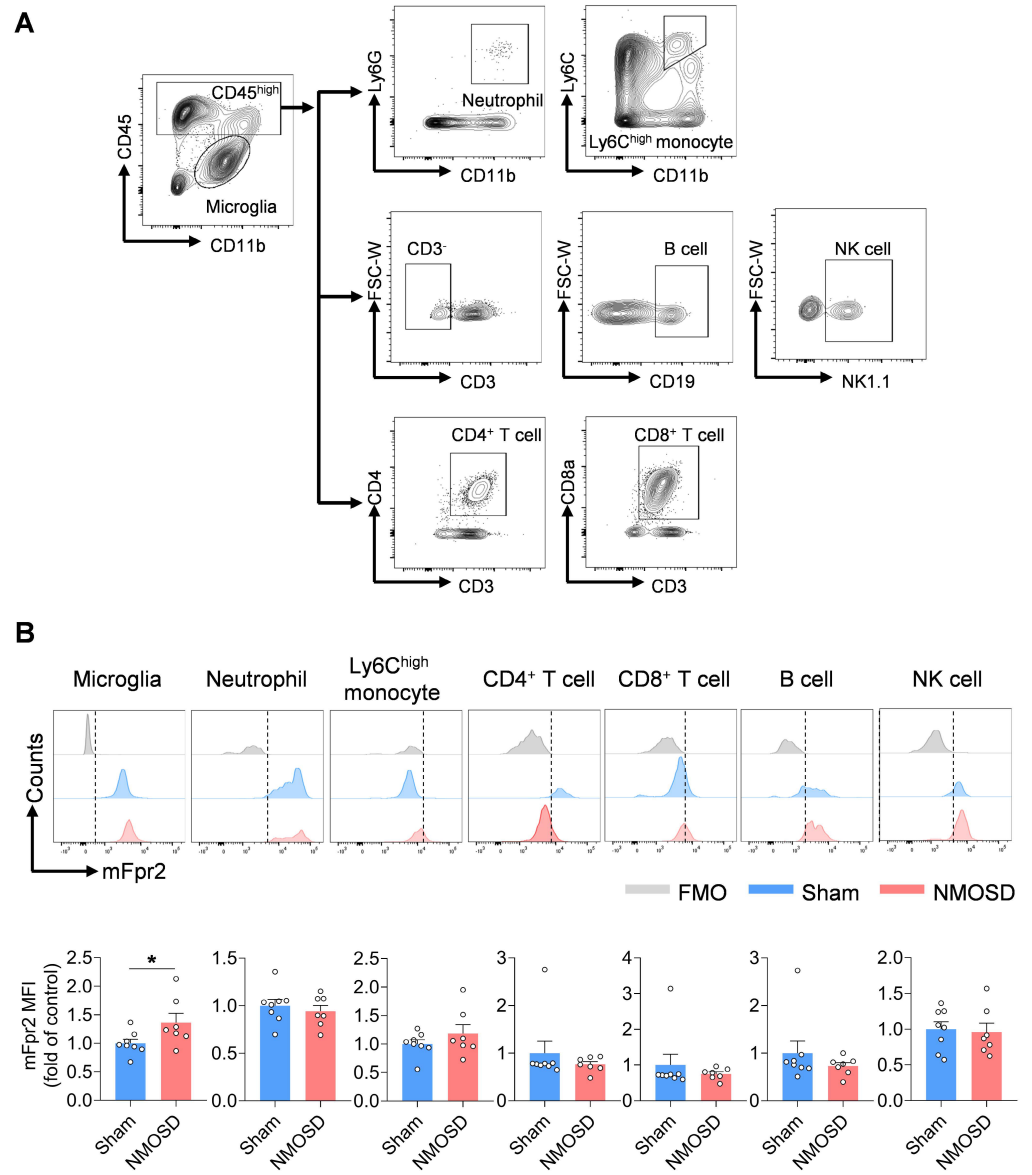
** Expression profile of mFpr2 in immune cell subsets from NMOSD mice.** (**A**) Gating strategy of microglia (CD11b^+^CD45^int^)**,** brain-infiltrating neutrophils (CD45^high^CD11b^+^Ly6G^+^), Ly6C^high^ monocytes (CD45^high^CD11b^+^Ly6C^high^), CD4^+^ T cells (CD45^high^CD3^+^CD4^+^), CD8^+^ T cells (CD45^high^CD3^+^CD8^+^), B cells (CD45^high^CD3^-^CD19^+^) and NK cells (CD45^high^CD3^-^NK1.1^+^). (**B**) Histograms showing mFpr2-expressing cell subsets in sham and NMOSD mice. Bar graphs showing the MFI of mFpr2 in microglia, neutrophils, Ly6C^high^ monocytes, CD4^+^ T cells and CD8^+^ T cells, B cells and NK cells in sham and NMOSD mice. Sham: n = 8, NMOSD mice: n = 7. Data are presented as mean ± SEM. *P < 0.05.

Next, we assessed the expression of mFpr2 across various immune cell types in the CNS using a mouse model of NMOSD induced by intracerebral injection of human complement (hC) and AQP4-IgG that was purified from AQP4-IgG-seropositive NMOSD patients. We found a remarkable upregulation of mFpr2 in the brain microglia of NMOSD mice as compared to sham controls or wild-type (WT) mice (**Figure 2A-B, Figure S1A-B**). In addition, immunostaining revealed that mFpr2 was expressed in microglia and neurons, along with a notable increase in mFpr2-expressing microglia following NMOSD induction (**Figure S2A-B**). Together, these results suggest that mFpr2 is mainly expressed in neutrophils, monocytes and microglia following NMOSD induction.

### FPR2 antagonism reduces NMOSD pathology in mice

**Figure 3:**
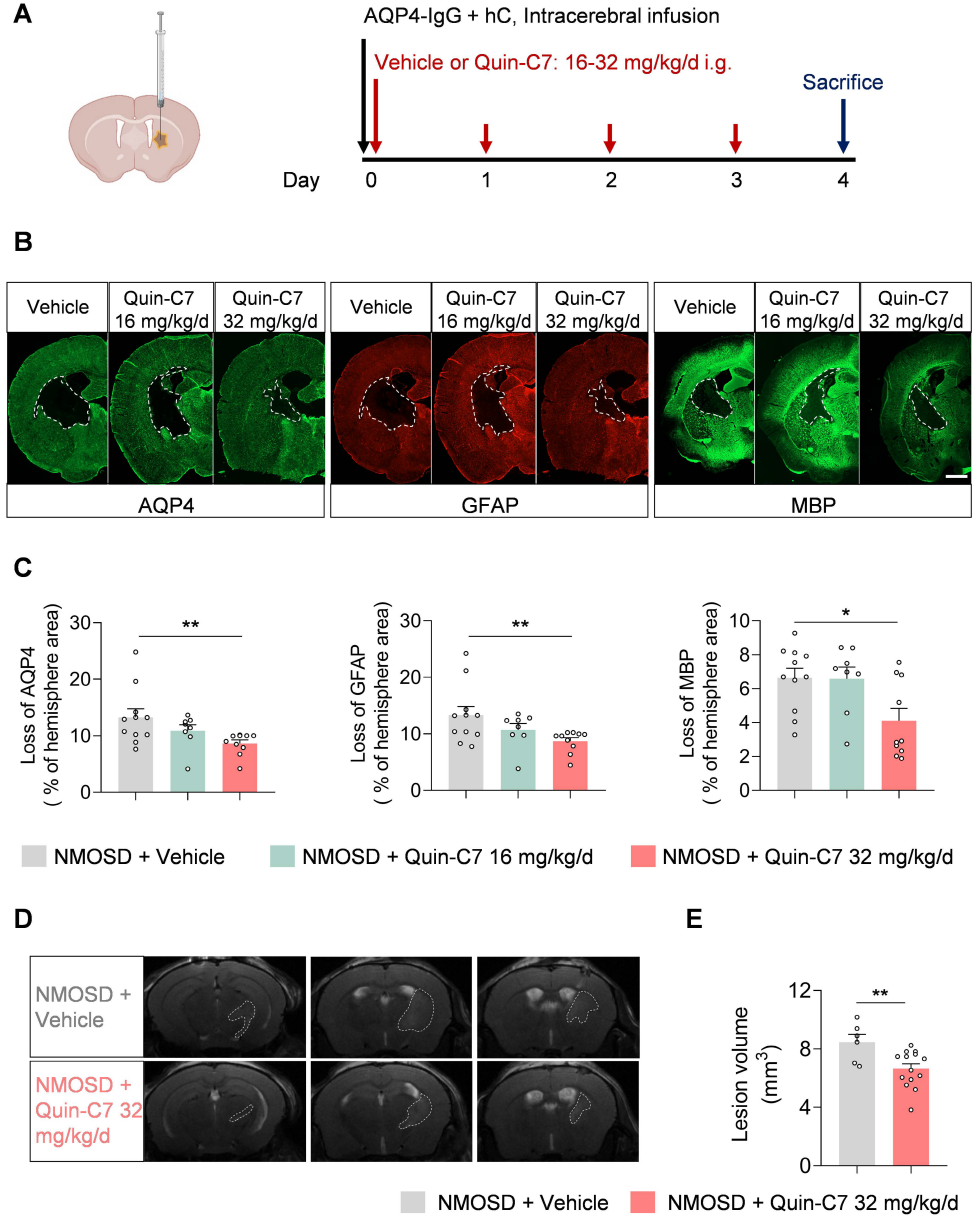
** FPR2 antagonism reduces NMOSD pathology in mice.** (**A**) Schematic diagram depicts experimental design. Mice received daily gavage administration of different dosages of Quin-C7 or an equal volume of vehicle for four consecutive days, starting immediately after NMOSD induction. Mouse brain tissue was collected for immunostaining analysis on day 4 post-NMOSD. (**B**) Immunostaining shows the expression of AQP4, GFAP, and MBP in indicated groups of NMOSD mice, white lines represent the area with the loss of AQP4, GFAP and MBP. Scale bar: 1mm. (**C**) Bar graphs showing the loss of AQP4, GFAP and MBP. n = 8-11 mice per group. (**D**) 9.4T-T2 weighted MR images showing brain lesions within the axial position in vehicle or Quin-C7-treated (32 mg/kg/day) NMOSD mice on day 4. (**E**) Bar graph showing the intracranial lesion volumes of the vehicle or Quin-C7-treated (32 mg/kg/day) NMOSD mice. Data are presented as mean ± SEM. *P < 0.05, **P < 0.01.

To investigate the role of mFpr2 in neuroinflammation and its impact on NMOSD pathology, we utilized a FPR2 antagonist Quin-C7 to block mFpr2 signaling^11^. NMOSD mice received daily treatment of Quin-C7 at different doses, starting immediately after NMOSD induction (**Figure 3A**). We found that FPR2 antagonism significantly reduced the loss of AQP4 (marker of astrocyte), glial fibrillary acidic protein (GFAP, marker of astrocyte activation), and myelin basic protein (MBP, marker of myelin sheath) at a dose of 32 mg/kg/day in NMOSD mice (**Figure 3B-C**), suggesting a decrease in both astrocyte damage and CNS demyelination in NMOSD mice. Additionally, we found a decrease in brain lesion volume in NMOSD mice receiving Quin-C7 (32 mg/kg/day) (**Figure 3D-E**). Notably, no lesion was found in groups of sham mice receiving Quin-C7 or vehicle control (**Figure S1C**). These results demonstrate that FPR2 antagonism using Quin-C7 reduces NMOSD pathology in mice.

### FPR2 antagonism reduces brain infiltration of lymphocytes and modulates the inflammatory activity of microglia in NMOSD mice

**Figure 4:**
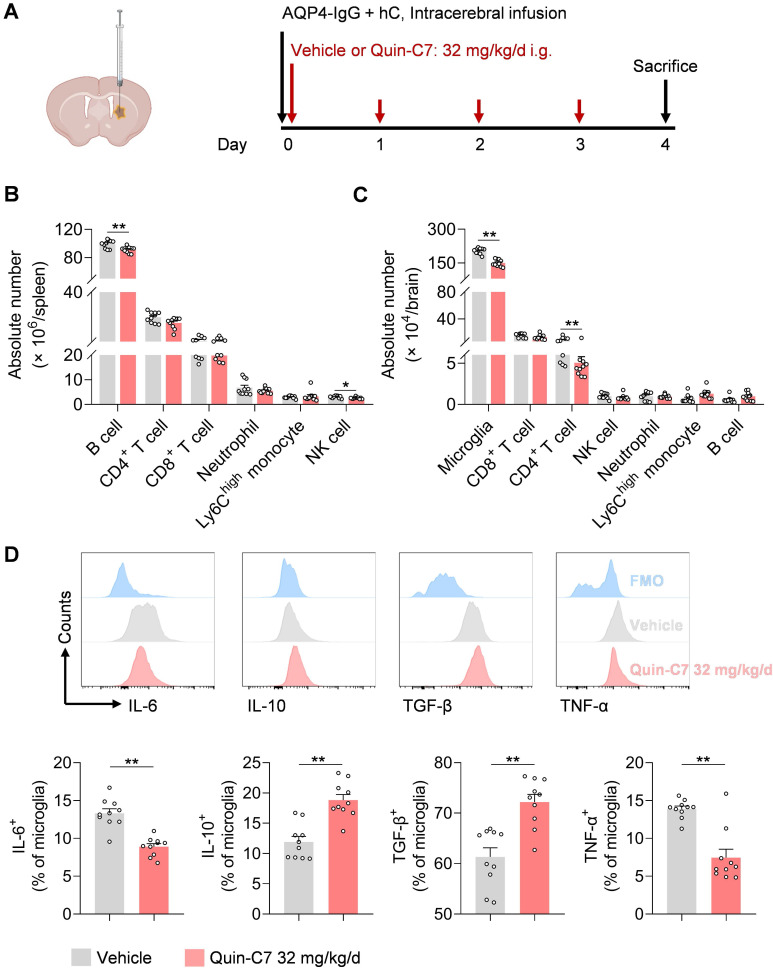
** FPR2 antagonism reduces CNS infiltration of lymphocytes and modulates microglia activity in NMOSD mice.** (**A**) Schematic diagram depicts experimental design. Mice received daily gavage administration of Quin-C7 (32 mg/kg/day) or an equal volume of vehicle for four consecutive days, starting immediately after NMOSD induction. Mouse brain and spleen tissues were collected for flow cytometry analysis on day 4 post-NMOSD. (**B**) Immune cell numbers in the spleen of vehicle or Quin-C7 (32 mg/kg/day) treated-NMOSD mice. (**C**) Immune cell numbers in the brain of vehicle or Quin-C7 (32 mg/kg/day) treated-NMOSD mice. (**D**) Flow cytometry analysis of IL-6, TNF-α, IL-10 and TGF-β in CD11b^+^CD45^int^ microglia from NMOSD mice receiving vehicle or Quin-C7 (32 mg/kg/day). Data are presented as mean ± SEM. *P < 0.05, **P < 0.01.

To examine the effects of FPR2 antagonism on immune cells in NMOSD, we performed flow cytometry to assess various immune cell types in the periphery and CNS following NMOSD induction in mice (**Figure 4A**). We found that FPR2 antagonism using Quin-C7 (32 mg/kg/day) reduced the number of B cells and NK cells in the spleen, as well as the number of microglia and infiltrating CD4^+^ T cells in the CNS (**Figure 4B-C**). Notably, similar counts of microglia and leukocyte subsets were seen in groups of sham controls receiving Quin-C7 or vehicle (**Figure S1D-E**). Additionally, FPR2 antagonism led to increased expression of anti-inflammatory factors (IL-10 and TGF-β), along with reduced expression of inflammatory factors (IL-6 and TNF-α) in microglia of NMOSD mice receiving Quin-C7 (**Figure 4D**). These results demonstrate that FPR2 antagonism suppresses lymphocyte infiltration into the brain and modulates microglia activity in NMOSD mice.

### Microglia contribute to the benefit of FPR2 antagonism in NMOSD mice

**Figure 5:**
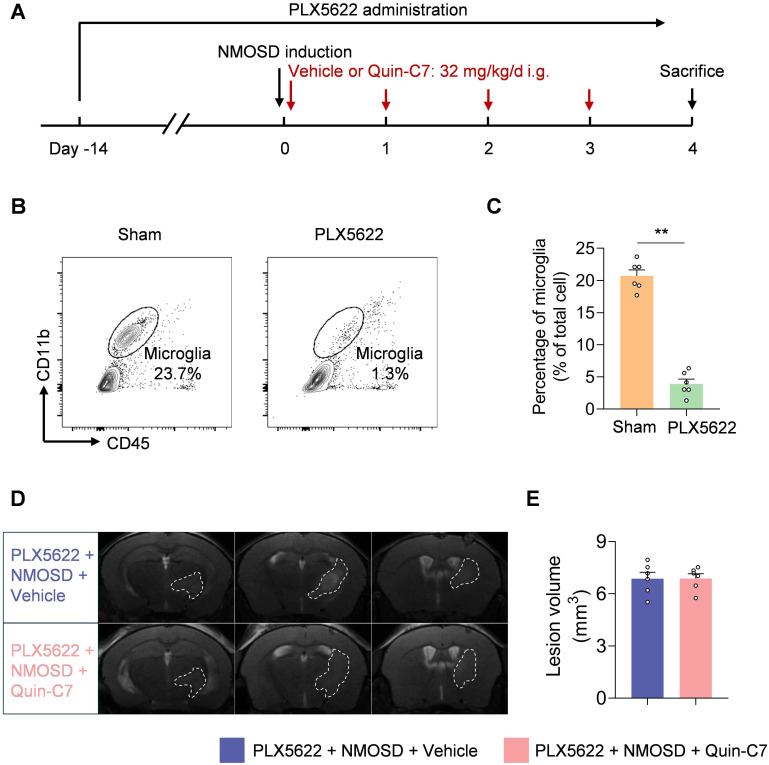
** Microglia contribute to the benefit of FPR2 antagonism in NMOSD mice.** (**A**) Schematic diagram illustrates drug administration and experimental design. C57BL/6 mice received oral gavage of PLX5622 for 14 days and continuously until the experiment ended. NMOSD was induced in PLX5622-treated mice by intracerebral injection of AQP4-IgG with hC. Thereafter, these mice received daily administration of Quin-C7 (32 mg/kg) or an equal volume of vehicle for 4 consecutive days starting immediately after NMOSD induction. On day 4 after NMOSD, the imaging analysis was performed. (**B-C**) Flow cytometry gating strategy (**B**) and bar graph (**C**) showing microglia percentage in the brain tissues obtained from mice receiving a control diet or PLX5622 for 14 days. n = 6 per group. (**D**) 9.4T-T2 weighted MR images showing brain lesions within the axial position in indicated groups of NMOSD mice. (**E**) Bar graph showing the volumes of intracranial lesions in indicated groups of NMOSD mice. Data are presented as mean ± SEM. **P < 0.01.

To determine the potential contribution of microglia to the protective effects of FPR2 antagonism in NMOSD, we used a colony-stimulating factor 1 receptor (CSF1R) inhibitor PLX5622 to deplete microglia (**Figure 5A**). In NMOSD mice receiving PLX5622, we found that >85% microglia were depleted (**Figure 5B-C**). Notably, FPR2 antagonism using Quin-C7 did not alter lesion volume in NMOSD mice receiving PLX5622 (**Figure 5D-E**), suggesting the involvement of microglia in observed benefit against NMOSD pathology in mice receiving FPR2 antagonist Quin-C7.

### T cells, B cells and NK cells are not involved in the benefit of FPR2 antagonism in NMOSD mice

**Figure 6:**
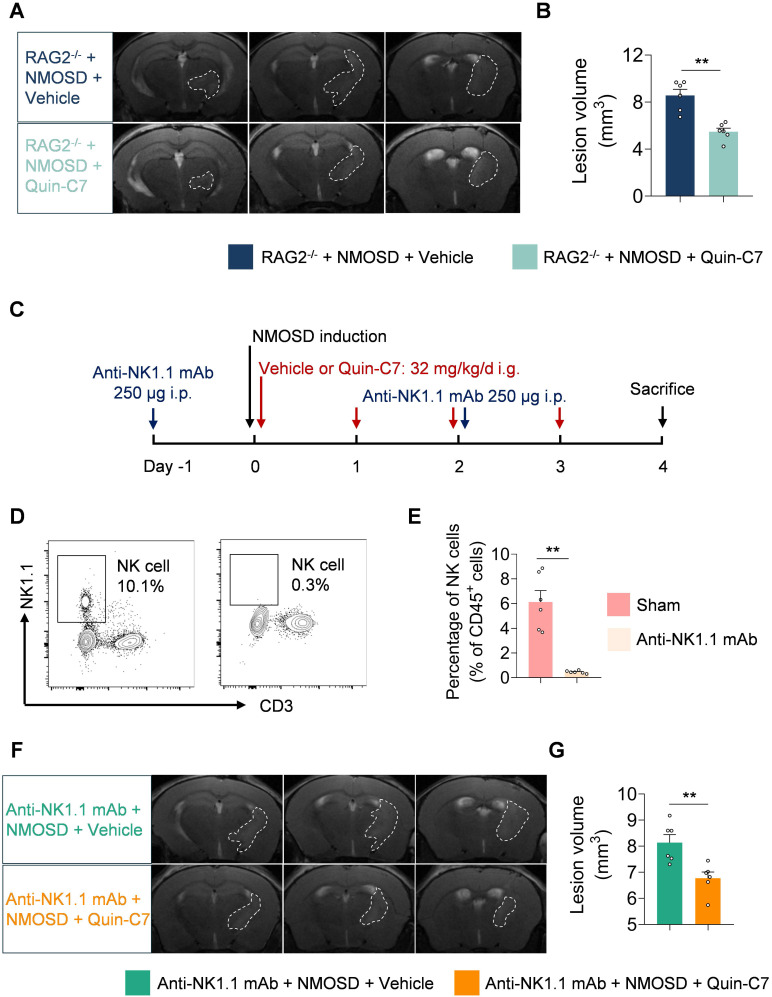
** T cells, B cells and NK cells are not involved in the benefit of FPR2 antagonism in NMOSD mice.** (**A**) 9.4T-T2 weighted MR images showing brain lesions within the axial position in RAG2^-/-^ NMOSD mice treated with vehicle or Quin-C7 (32 mg/kg/day). (**B**) Bar graph showing the volumes of intracranial lesions in RAG2^-/-^ NMOSD mice treated with vehicle or Quin-C7 (32 mg/kg/day). (**C**) Schematic diagram depicts experimental design. NMOSD was induced in NK depleted mice by intracerebral injection of AQP4-IgG with hC. Thereafter, these mice received daily administration of Quin-C7 (32 mg/kg) or an equal volume of vehicle for 4 consecutive days starting immediately after NMOSD induction. On day 4 after NMOSD, the imaging analysis was performed. For NK cell depletion, mice were injected i.p. with 250 μg anti-NK1.1 mAb or isotype control every three days, the first time was injected one day before NMOSD induction. (**D-E**) Flow cytometry gating strategy (**D**) and quantification of NK cell percentage (**E**) in the peripheral blood of mice with or without anti-NK1.1 mAb treatment. n = 6 per group. (**F**) Representative 9.4T-T2 weighted MR images showing brain lesions within the axial position in indicated groups of NMOSD mice. (**G**) The histogram shows the volumes of intracranial lesions in indicated groups of NMOSD mice. Data are presented as mean ± SEM. **P < 0.01.

To investigate the potential role and function of lymphocytes in the protective effects of FPR2 antagonism in NMOSD, we utilized RAG2^-/-^ mice in which T and B cells are devoid. We found that FPR2 antagonism using Quin-C7 reduced lesion volume in RAG2^-/-^ NMOSD mice (**Figure 6A-B**), suggesting that the benefit of FPR2 antagonism against NMOSD pathology may not involve T and B cells.

To evaluate whether NK cells are involved in the protective effects of FPR2 antagonism in NMOSD, we used anti-NK1.1 monoclonal antibody (mAb; clone: PK136) to deplete NK cells (**Figure 6C**). We found that anti-NK1.1 mAb eliminated more than 90% of NK cells (**Figure 6D-E**) and FPR2 antagonism using Quin-C7 reduced lesion volume in NMOSD mice receiving anti-NK1.1 mAb (**Figures 6F-G**), suggesting that the benefit of FPR2 antagonism against NMOSD pathology may not involve NK cells.

## Discussion

This study identified FPR2 as a master switch in control of neuroinflammation and NMOSD pathology. As documented here, we found that FPR2/mFpr2 was mainly expressed in microglia within the CNS and various immune cell subsets including myeloid cells and lymphocytes in the periphery in patients with NMOSD and its mouse model. FPR2 antagonism using an antagonist Quin-C7 led to reduced brain lesion volume, astrocyte loss, and CNS demyelination in NMOSD, accompanied by reduced inflammatory activity of microglia, and reduced lymphocytes infiltration both in the spleen and brain. Notably, the protective effects of FPR2 antagonism mainly involve microglia but not T cells, B cells and NK cells. Collectively, our findings revealed a previously unrecognized role of FPR2 in CNS inflammation, suggesting that targeting FPR2 could serve as a novel treatment option for future design of immune therapies for NMOSD and perhaps other neuroimmune disorders.

As early responders to CNS insults, microglia drive CNS inflammatory cascades and demyelination in NMOSD both in animal models and patients with NMOSD^12^-^14^. We found that FPR2 antagonism reduced microglia activation, augmented their anti-inflammatory activity as reflected by increased production of IL-10 and TGF-β, along with reduced production of IL-6 and TNF-α, suggesting the beneficial effects of FPR2 antagonism involves its impact on microglia. This view is supported by the finding that depletion of microglia abolished the protective effects of FPR2 antagonism. In support of our findings, previous studies demonstrated that FPR2 modulation suppressed the inflammatory activity of myeloid cells^9^, ^15^, ^16^. Mechanistically, the impact of FPR2 antagonism on microglia may involve its effects to prevent the detrimental activation of FPR2 when exposed to its pro-inflammatory ligands including but not limited to serum amyloid A, prion protein and annexin A1, etc.^17^, ^18^. Therefore, it is reasonable to postulate that FPR2 antagonism-induced modulation of microglia activity may result from reduced FPR2 stimulation by danger signals released from the injured CNS.

Although mainly expressed by myeloid cells in the arm of innate immunity^19^-^21^, FPR2 not only controls myeloid cell activity such as microglia but also modulates the activity of lymphocytes in adaptive immunity. Reportedly, genetic deficiency of the resolvin D1 receptor FPR2 led to dysfunction of T cell activity^22^. A previous study also revealed that FPR2 modulation affects Th17 cell differentiation in experimental autoimmune encephalomyelitis^8^. Of interest, we observed that FPR2 antagonism reduced B cells and NK cells in the spleen, as well as CNS infiltration of CD4^+^ T cells in NMOSD mice, although genetic deficiency or antibody depletion of lymphocytes did not affect the benefits of FPR2 antagonism in NMOSD. These results suggest that the effects of FPR2 antagonism on lymphocyte activity and their entry into CNS may indirectly result from its impact on microglia, which warrant future investigations.

Previous studies revealed that FPR2 modulates neutrophil extravasation, apoptosis and efferocytosis^17^, ^19^, ^21^, ^23^, ^24^. In our study, although FPR2 is highly expressed in neutrophils, FPR2 antagonism did not significantly alter the number and activity of neutrophils in the periphery and neutrophil infiltration into CNS in NMOSD mice, suggesting that FPR2 antagonism may not significantly affect neutrophil function and their contribution to NMOSD pathology. We postulate that this is probably caused by relatively low levels of FPR2 ligands in the periphery that are released from the injured CNS in NMOSD mice because the lesion volume and cell death are relatively limited in this model. Nevertheless, the effects of FRP2 antagonism on neutrophils and their contribution to NMOSD pathology require future studies. We also acknowledge the differences between the immune profile in human NMOSD and our mouse NMOSD model, which may not fully capture the complexity of the disease. Despite the absence of increased neutrophils, B cells and NK cells in this NMOSD model, FPR2 antagonism remained effective, and microglia are involved in the observed protective effects. Future studies are required to address the limitations of this model.

In summary, our findings demonstrate an immunoregulatory role of FPR2 antagonism to restrict detrimental neuroinflammation and NMOSD pathology. The beneficial effects of FPR2 antagonism mainly involve microglia but not lymphocytes. These findings indicated a previously unrecognized role of FPR2 in NMOSD and deserve future investigations of its potential clinical implications in neuroimmune disorders.

## Materials and methods

### Human blood sample

Peripheral blood samples for scRNA-seq were collected from 5 treatment-naïve NMOSD patients (1 male, 4 female) and 5 healthy controls (2 male, 3 female). There was no significant difference in the age of the recruited subjects (NMOSD vs. Control: 41.8 ± 9.5 vs. 47.4 ± 13.6 years, *P* = 0.519). Blood samples for flow cytometry were collected from 10 NMOSD patients (1 male, 9 female) and 20 healthy controls (8 male, 12 female). There was no significant difference in the age of the recruited subjects (NMOSD vs. Control: 34.5 ± 12.2 vs. 33.7 ± 5.4 years, *P* = 0.94). The study protocol was approved and conducted in accordance with the Declaration of Helsinki.

### scRNA-seq data analysis

Single-cell suspensions of peripheral blood mononuclear cells from 5 patients with NMOSD and 5 control subjects were subjected to scRNA-seq using 10× Genomics. After processing with CellRanger, the gene count matrix of each sample was combined for downstream analysis. Subsequent quality control steps retained 90,381 single cells, and the data were integrated, subjected to dimensionality reduction, clustered, and visualized. Seurat was used for these processes. Cell counts were normalized and converted to the log scale using the NormalizedData function and scaled with the ScaleData function. Principal component analysis (PCA) was performed, and the Harmony v1 integration method was applied to correct for potential batch effects. The top 30 principal components and a 'resolution = 0.8' were used for clustering. Cell cluster identities were assigned based on previously reported cell-type-specific markers^10^, including neutrophils (FCGR3B, NAMPT), monocytes (CD14, FCGR3A), B cells (CD79A, CD19, MS4A1), plasma cells (MZB1), CD4^+^ T cells (CD3D, CD4), CD8^+^ T cells (CD3D, CD8A), and natural killer cells (NK cells; KLRF1, NKG7, GNLY).

### Animal

C57BL/6 mice (8-10 weeks of age) were purchased from Spafford Laboratories (Beijing, China). RAG2^-/-^ mice were purchased from Huafukang Bioscience (Beijing, China). All mice were bred on a C57BL/6 background and maintained under pathogen-free conditions. The mice used in this study were housed in groups of no more than five per cage, under a standardized light-dark cycle, with ad libitum access to food and water. The vivarium was maintained at a controlled temperature of 21±1°C and humidity ranging from 50% to 60%.

### AQP4-IgG isolation and hC preparation

Serum samples were collected from patients with NMOSD who had AQP4-IgG seropositivity. IgG was purified from the serum using a protocol involving protein G-agarose, as previously described^25^. The final IgG concentration was 8.0 mg/ml. For the healthy control group, IgGs were obtained from age-matched healthy volunteers using the same protocol, with a final concentration of 8.0 mg/ml. Human complement (hC) was collected as described^25^.

### NMOSD mouse model

The NMOSD model was induced based on a previous study with some modifications^25^. Briefly, mice were anesthetized by inhaling 3% isoflurane and maintained under anesthesia with continuous inhalation of 1% isoflurane. Subsequently, the mice were mounted onto a stereotactic frame (RWD Life Science, Shenzhen, China). A midline scalp incision was made to expose the bregma, and a burr hole was drilled on the right side of the skull manually, positioning 2.5 mm lateral to the bregma. A mixture of AQP4-IgG and hC in a 6:4 ratio was prepared and resuspended. A 50-μl microsyringe fitted with a 34-gauge needle (Hamilton) was inserted 3 mm into the brain parenchyma, and 10-μl of the mixed solution was then infused into the brain. Finally, the scalp was sutured after disinfecting the surgical site.

### Drug administration

To determine the effects and optimal dosage of Quin-C7 in NMOSD, Quin-C7 was administered immediately following NMOSD induction. Quin-C7 was dissolved in a solution containing DMSO and 2% P188, with a DMSO-to-P188 ratio of 1:99. Mice were given Quin-C7 at doses of 16 mg/kg or 32 mg/kg by oral gavage daily after NMOSD induction. Mice receiving an equal volume of vehicle (DMSO + 2% P188 with ratio of 1:99) were designated as controls.

To deplete microglia, PLX5622 (Selleckchem, Houston, TX, USA) was formulated into AIN-76A standard chow at a dose of 1.2 g PLX5622 per kilogram of diet. Six-week-old mice were fed this chow containing either PLX5622 or control diet for 14 consecutive days prior to NMOSD induction. Drug treatment continued throughout the experiment. For NK cell depletion, anti-mouse NK1.1 monoclonal antibody (mAb, Clone: PK136; BioXcell, West Lebanon, NH, USA) was administered by intraperitoneal injection every 3 days at a dose of 250 µg per mouse, starting 1 day before NMOSD induction.

### Magnetic resonance imaging

Lesion volume was quantified on day 4 after NMOSD induction using a 9.4T small animal MRI. T2-weighted images were acquired to assess lesion volume. The imaging parameters were as follows: repetition time (TR) = 5100 ms, effective echo time (TE) = 31.71 ms, inversion time (TI) = 2506.097 ms, and slice thickness = 0.50 mm. The lesion volumes were manually outlined, and the total volume was calculated by multiplying the sum of the areas by the inter-slice distance (0.50 mm). Two investigators, blinded to the experimental groups, independently calculated the lesion volumes.

### Immunostaining

Brain tissue was collected from mice on day 4 post-NMOSD induction and incubated overnight at 4°C with the following primary antibodies: Iba1 (1:500, NB100-1028, Novus Biologicals, Littleton, CO, USA), AQP4 (1:200, PB9475, Boster, Wuhan, Hubei, China), GFAP (1:500, 14-9892-82, Invitrogen, Carlsbad, CA, USA), and MBP (1:500, 78896S, CST, Danvers, MA, USA), FPR2 (1:200, ab203129, Abcam, Cambridge, UK). The tissue was then incubated with fluorochrome-conjugated secondary antibodies at room temperature for 1.5 hours: donkey anti-rabbit 488 (1:1000, A32790, Invitrogen, Carlsbad, CA, USA), donkey anti-goat 546 (1:1000, A11056, Invitrogen, Carlsbad, CA, USA) or goat anti-mouse 546 (1:1000, A11030, Invitrogen, Carlsbad, CA, USA). Slides were mounted using DAPI Fluoromount-G (Southern Biotech, Birmingham, AL, USA). Images were captured using fluorescence microscopy (BioTek, Winooski, VT, USA) and analyzed with ImageJ software.

### Flow cytometry

Peripheral blood samples were collected from patients with NMOSD and healthy control subjects. Peripheral blood mononuclear cells (PBMCs) were isolated from whole blood specimens and subsequently stained for flow cytometry analysis. Peripheral blood immune cells, including neutrophils (CD45^+^CD16^+^ CD15^+^CD66b^+^), monocytes (CD45^+^CD16^-^CD14^+^), B cells (CD45^+^CD3^-^CD19^+^), CD4^+^ T cells (CD45^+^CD3^+^ CD4^+^), CD8^+^ T cells (CD45^+^CD3^+^CD8^+^) and NK cells (CD45^+^CD3^-^CD56^+^) , were characterized and evaluated for FPR2 expression using a PE-conjugated anti-human antibody against FPR2 (1:100, R&D Systems, Minneapolis, MN, USA).

Single-cell suspensions from mouse spleen or brain tissues were prepared as previously described^26^. To quantify cells expressing mFpr2 and evaluate the effects of Quin-C7 on peripheral and CNS immune cells in NMOSD mice, we focused on the following cell populations: microglia (CD11b^+^CD45^int^), and brain-infiltrating neutrophils (CD11b^+^CD45^high^ Ly6G^+^), Ly6C^high^ monocytes (CD11b^+^CD45^high^Ly6C^high^), CD4^+^ T cells (CD45^high^CD3^+^CD4^+^), CD8^+^ T cells (CD45^high^CD3^+^CD8^+^), B cells (CD45^high^CD3^-^CD19^+^), and NK cells (CD45^high^CD3^-^NK1.1^+^); In addition, we assessed peripheral immune cell populations, including spleen neutrophils (CD45^+^CD11b^+^Ly6G^+^), Ly6C^high^ monocytes (CD45^+^ CD11b^+^Ly6C^high^), CD4^+^ T cells (CD45^+^CD3^+^CD4^+^), CD8^+^ T cells (CD45^+^CD3^+^CD8^+^), B cells (CD45^+^CD3^-^ CD19^+^), NK cells (CD45^+^CD3^-^NK1.1^+^), by isolating cellular components to perform flow cytometry analysis.

The expression of mFpr2 was assessed using a rabbit anti-FPR2 primary antibody (1:50, ab203129, Abcam, Cambridge, UK; ab203129, Abcam, Cambridge, UK), followed by an AF647-conjugated anti-rabbit secondary antibody for detection. For intracellular cytokine staining (IL-6, IL-10, TNF-α, and TGF-β produced by microglia), single cells were stimulated for 4 hours in the presence of Cell Activation Cocktail with Brefeldin A (BioLegend, San Diego, CA, USA) and then harvested for surface and intracellular staining.

### Statistical analysis

Power analysis was performed using SAS 9.1 software (SAS Institute Inc.). Sample size was based on our experience with the respective tests, variability of the assays and inter-individual differences among experimental groups. The experimental design was based on our prior publications with similar mechanistic studies completed in our laboratory. Animals were randomly assigned to experimental groups, based on the random number generator function in Microsoft Excel. All experiments presented in this study were repeated at least three times. A two-tailed unpaired Student's *t*-test was used to compare data from two independent groups. For comparison of two or more variables among multiple groups, two-way ANOVA followed by Tukey post hoc test was used. *P* < 0.05 was considered to be significant. Data were analyzed with GraphPad prism 8.0. All data were presented as mean ± SEM.

## Supplementary Material

Supplementary figures.
