# Supplementary material for: Targeting formyl peptide receptor 2 to suppress neuroinflammation in neuromyelitis optica spectrum disorder
Source: Theranostics. 2025 Mar 19;15(10):4495–506. doi: 10.7150/thno.107303 (PMC11984409; doi:10.7150/thno.107303)
Supplement: Supplementary file 1 — Supplementary figures. [file thnov15p4495s1.pdf]

Supplementary Figures

Figure S1. Effects of Quin-C7 treatment on mFpr2 expression and CNS inflammation in groups of sham control mice.

(**A**) Histograms showing mFpr2-expressing microglia in WT, sham + vehicle, and sham + Quin-C7 (32 mg/kg/day) mice. (**B**) Bar graphs showing the MFI of mFpr2 in microglia between WT, sham + vehicle, and sham + Quin-C7 (32 mg/kg/day) mice. (**C**) 9.4T-T2 weighted MR images showing brain lesions within the axial position in sham + vehicle, and sham + Quin-C7 (32 mg/kg/day) mice on day 4. (**D-E**) Immune cell numbers in the brain (**D**) and spleen (**E**) of vehicle or 32 mg/kg/day Quin-C7 treated-sham mice. n = 6 mice per group. Data are presented as mean ± SEM.

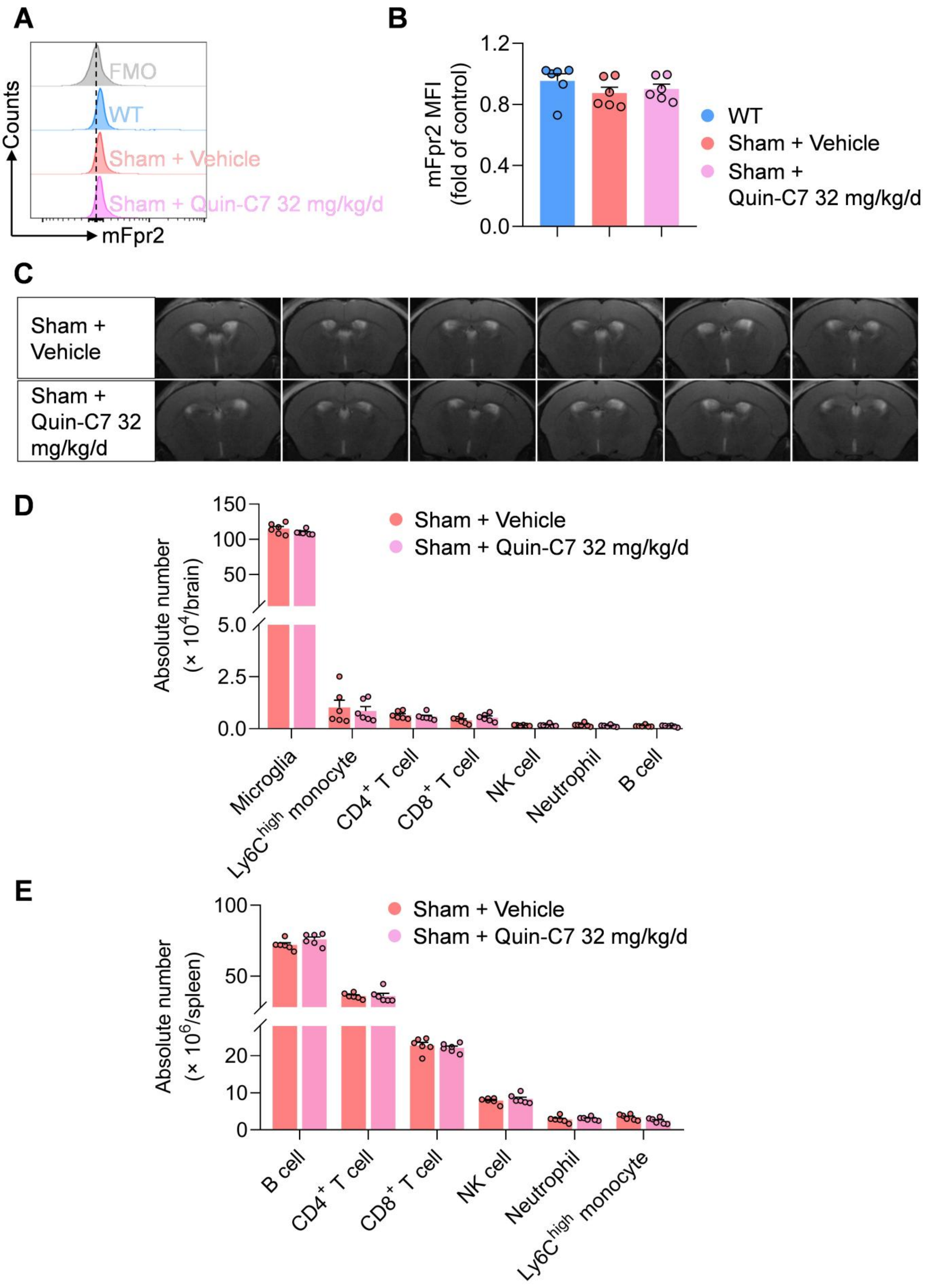

A
FMO
WT
Sham + Vehicle
Sham + Quin-C7 32 mg/kg/d
Counts
mFpr2
B
mFpr2 MFI (fold of control)
1.2
0.8
0.4
0.0
WT
Sham + Vehicle
Sham + Quin-C7 32 mg/kg/d
C
Sham + Vehicle
Sham + Quin-C7 32 mg/kg/d
D
Absolute number (× 10^4/brain)
150
100
50
5.0
2.5
0.0
Sham + Vehicle
Sham + Quin-C7 32 mg/kg/d
Microglia
Ly6C^high monocyte
CD4^+ T cell
CD8^+ T cell
NK cell
Neutrophil
B cell
E
Absolute number (× 10^6/spleen)
100
50
20
10
0
Sham + Vehicle
Sham + Quin-C7 32 mg/kg/d
B cell
CD4^+ T cell
CD8^+ T cell
NK cell
Neutrophil
Ly6C^high monocyte

Figure S2. Immunostaining of mFpr2-expressing brain-resident cells in NMOSD mice.

Brain sections from sham or NMOSD mice were stained with mFpr2, in combination with Iba1, NeuN, or GFAP, to assess mFpr2-expressing microglia, neurons, or astrocytes, respectively. (**A**) Immunostaining images showing mFpr2-expressing microglia, neurons and astrocytes on day 4 in groups of sham and NMOSD mice. (**B**) Bar graphs showing the number of mFpr2-expressing microglia, neurons or astrocytes on day 4 in sham and NMOSD mice. Scale bar, 100 μm. n = 6 mice per group. Data were presented as mean ± SEM, $^{**}P < 0.01$.

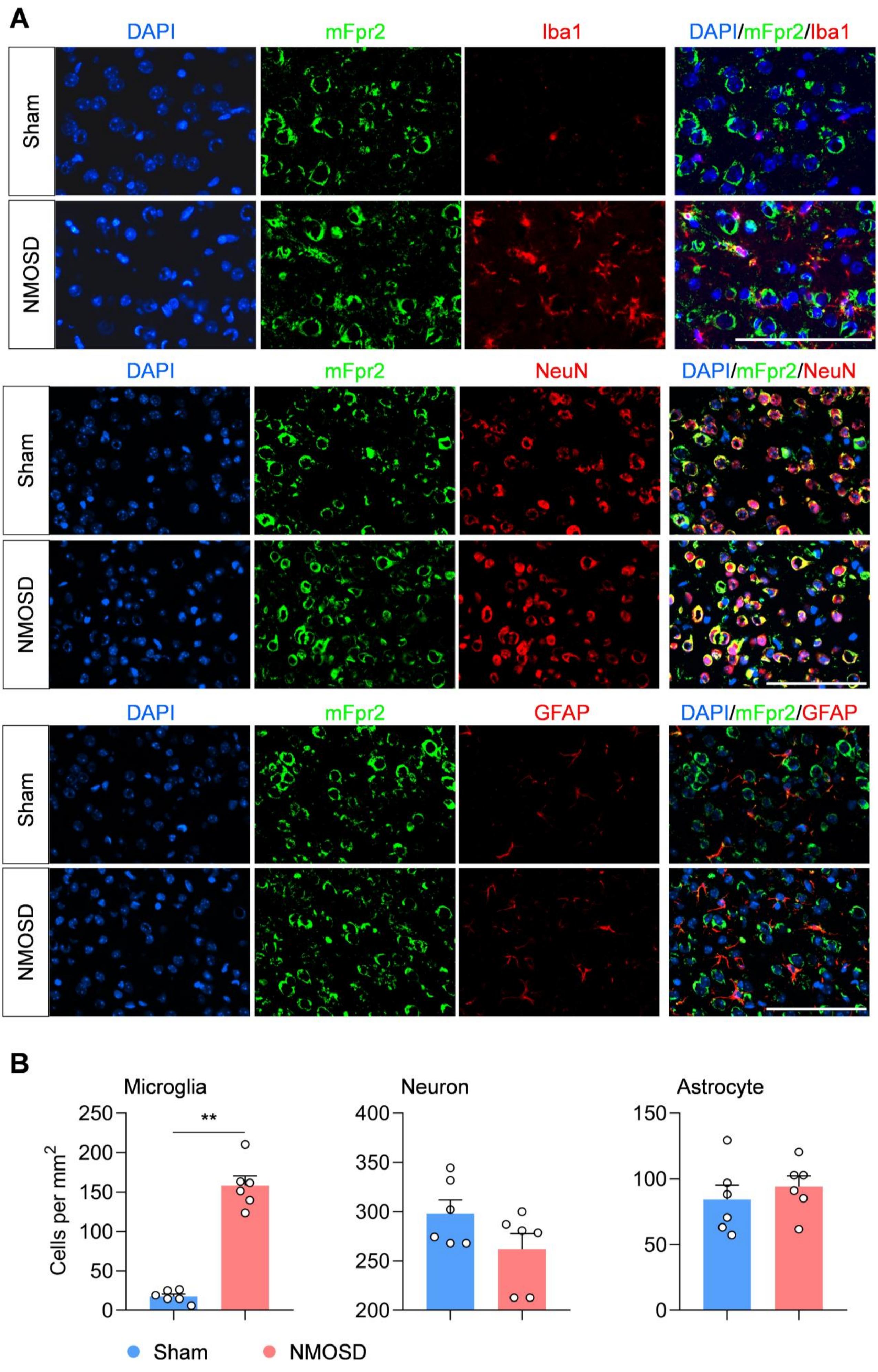
A
DAPI
mFpr2
Iba1
DAPI/mFpr2/Iba1
Sham
NMOSD
DAPI
mFpr2
NeuN
DAPI/mFpr2/NeuN
Sham
NMOSD
DAPI
mFpr2
GFAP
DAPI/mFpr2/GFAP
Sham
NMOSD
B
Microglia
Neuron
Astrocyte
Cells per mm2
**
250
200
150
100
50
0
400
350
300
250
200
150
100
50
0
Sham
NMOSD
